# Analysis and comparison of physiochemical properties, mutations and glycosylation patterns between RNA polymerase and membrane protein of SARS-CoV and SARS-CoV-2

**DOI:** 10.22099/mbrc.2021.42187.1692

**Published:** 2021-12

**Authors:** Mandana Behbahani, Parisa Rabiei, Hassan Mohabatkar

**Affiliations:** Department of Biotechnology, Faculty of Biological Science and Technology, University of Isfahan, Isfahan, Iran

**Keywords:** COVID-19, Betacoronavirus, MEME motifs, Glycosylation, Substitution mutation

## Abstract

SARS-CoV-2 is a member of β-genus of the coronavirus subfamily, alongside the virus that causes SARS (Severe Acute Respiratory Syndrome). As implied by their names, SARS-CoV-2 and SARS-CoV genome sequences have close kinship (about 79% genomic sequence similarity). In the current research, sequence-based physiochemical properties of RNA polymerase and membrane glycoprotein of SARS-CoV-2 and SARS-CoV were compared. In addition, impacts of substitution mutations on stability and glycosylation patterns of these proteins were studied. In comparison of physiochemical features of membrane and RNA polymerase proteins, only instability index of membrane protein was difference between SARS-CoV and SARS-CoV-2. Mutation analysis showed increase in stability of RNA polymerase and decrease in stability of membrane protein in SARS-CoV-2. Glycosylation pattern analysis showed glycosylation enhancement in both membrane and RNA polymerase proteins of SARS-CoV-2 in comparison to SARS-CoV. In conclusion, more glycosylation and stability of SARS-CoV-2 RNA polymerase could be one of the reasons of high pathogenicity property and host immune system evasion of SARS-CoV-2.

## INTRODUCTION

SARS-CoV-2 belongs to the Betacoronavirus genus and causes severe respiratory disease in humans. Other viruses in this family are SARS and MERS coronaviruses. SARS-CoV-2 genome sequence has about 79% and 50% sequence similarity to SARS-CoV and MERS-CoV respectively [1-3]. Structurally, four main structural proteins and several accessory proteins are observed in SARS-CoV-2. Main proteins include spike (S) glycoprotein, small envelope (E) glycoprotein, membrane (M) glycoprotein, and nucleocapsid (N) protein [4]. In the virus life cycle, membrane glycoprotein has a key role in binding to other structural proteins and stabilizing of nucleocapsid protein-RNA complex which is crucial for promoting completion of viral assembly [5]. This protein also interacts with envelope E protein in the budding compartment of the host cell, which is located between endoplasmic reticulum and the Golgi complex. SARS-CoV membrane protein is an important protein because it may function as a cytosolic pathogen-associated molecular pattern to stimulate IFN-β production by activating a Toll-like receptor-related TRAF3-independent signaling cascade [6] . 

Among nonstructural proteins of SARS-CoV-2, RNA-dependent RNA polymerase plays a central role in the virus life cycle [7]. RNA polymerase is encoded by all RNA viruses and some DNA viruses with various sequence motifs and tertiary structures [8-10]. SARS-CoV-2 RNA polymerase is derived from proteolytic processing of polyprotein precursors [11]. 

Since comparison studies of SARS-CoV-2 with other members of Betacoronavirus genus can pave the way toward understanding more details about this virus properties and its behavior in the body, an in silico comparison was carried out in this work. In the present study, physicochemical properties, mutation sites and glycosylated positions of RNA polymerase and membrane protein of SARS-CoV and SARS-CoV-2 have been compared. 

## MATERIALS AND METHODS

**Data collection and MEME motif discovery: **Amino acid sequences of membrane and RNA polymerase proteins of SARS-CoV-2 and SARS-CoV were fetched from NCBI. Accession number of proteins and their sequence length are shown in Table 1. The motifs of these proteins were obtained via MEME motif discovery webserver [12]. Factors of MEME were applied as following: minimum width for each motif, six; maximum width for each motif, fifty; maximum number of motifs to discover three and amount of each motif, zero or one per sequence.

**Table 1:** Accession number of proteins related to SARS-CoV and SARS-CoV-2

**Type of protein**	**SARS CoV**	**Length (aa)**	**SARS CoV2**	**Length (aa)**
**Membrane protein**	AAT76152.1	221	QIA98586.1	222
**RNA polymerase**	ATO98167.1 ATO98179.1 AID 16712.1 AID 16714.1	932 932 932 932	YP_009725307.1	932

**Physicochemical properties analysis: **ProtParam available at http://web.expasy.org/ protparam is a prediction tool, which calculates physicochemical properties of proteins [13-16]. In this study, four characteristics (theoretical pI, extinction coefficient, aliphatic index, grand average of hydropathicity and instability) of membrane glycoprotein and RNA polymerase of SARS-CoV-2 and SARS-CoV and their motifs were evaluated using ProtParam. 

**Mutation discovery and stability effects analysis: **To find amino acid differences between SARS-CoV-2 and SARS-CoV proteins, protein-protein pairwise alignment tool available in NCBI was used. Then, protein stability changes upon single point mutation were predicted using I-Mutant-2.0 webserver (https://folding.biofold.org/i-mutant/i-mutant2.0.html) for membrane and RNA polymerase proteins separately [17]. I-Mutant is a robust prediction tool that can predict protein stability changes through protein structure or more importantly protein sequence [17, 18]. This server only could take into account amino acid substitution mutations and insertion and deletion mutations could not be covered. SARS-CoV protein sequences were inputted in I-Mutant server. Then, “position” and “new residue” boxes were filled by location number and amino acid type of occurred substitution mutations in SARS-CoV-2 sequence rather than SARS-CoV protein sequences. The prediction was carried out based on free energy change value (DDG) at pH value of 7 and room temperature.

**Function changes upon mutation analysis: **To analyze mutation effects on function of desired proteins, the sorting intolerant from tolerant (SIFT) algorithm [19] was used. SIFT webserver is able to predict substitution mutations on amino acids which can likely change the function of a protein [20]. This algorithm works based on sequence homology and the physico-chemical similarity between the alternate amino acids [21, 22]. SARS-CoV membrane and RNA polymerase protein sequences were inputted to SIFT server and a table of scaled probabilities for entire proteins were achieved for two proteins individually.

**Glycosylation prediction: **Among various post-translational modifications, glycosylation is critically associated with pathogenicity strength, immune evasion and host-pathogen interactions and has main influence on activity, conformation and stability of a protein [23, 24]. In this study, N-linked and O-linked glycosylation sites of whole membrane protein and RNA polymerase were predicted using their sequences by means of GPP webserver [25] (https://comp.chem.nottingham.ac.uk/home/index.html), and then results were compared between SARS-CoV and SARS-CoV-2. 

## RESULTS

**Figure 1:**
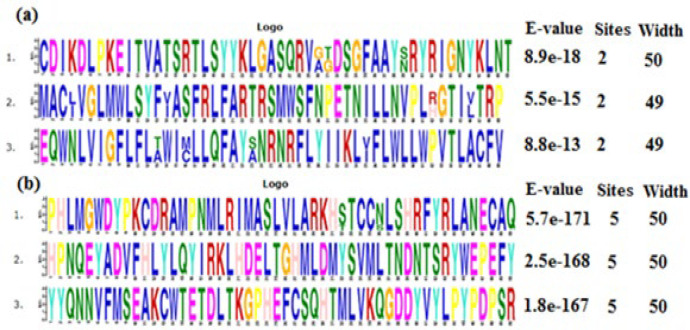
Comparison of three most probable motifs discovered by MEME motif discovery webserver for a) Membrane protein of SARS-CoV and SARS-CoV-2 viruses; b) RNA polymerase protein of SARS-CoV and SARS-CoV-2 viruses. The motifs with closer e-value to zero are more valuable

In this bioinformatics study, two main proteins of SARS-CoV-2, membrane and RNA polymerase, were compared with their analogous proteins in SARS-CoV in aspects of physiochemical properties and effect of point mutations on their function. The results of MEME webserver revealed three SARS-CoV (158-208, 83-132, 17-66) and three SARS-CoV-2 (159-209, 84-133,18-67) motifs for membrane protein. Additionally, three SARS-CoV (4981-5030, 5156-5205, 5241-5290) and three SARS-CoV-2 (612-661, 787-836, 872-921) motifs were indicated for RNA polymerase protein (Fig. 1). 

Outcomes of membrane proteins and their motifs analysis by ProtParam webserver showed that stability index of membrane glycoprotein of SARS-CoV-2 and their motifs were significantly more than SARS-CoV (Table 2). However, molecular weight, isoelectric point, aliphatic and extinction coefficient of these proteins and their motifs were not significantly different. 

**Table 2:** Physicochemical properties of whole protein and most probable motifs of Membrane glycoprotein of SARS-CoV-2 and SARS-CoV

**Motif**	**Protein**	**Aliphatic**	**Instability**	**PI**	**Extension Coefficient**	**GRAVY**
-	Membrane glycoprotein SARS-CoV-2	120.86	39.14	9.51	52160	0.446
-	Membrane glycoprotein SARS-CoV	116.08	28.58	9.81	52035	0.415
Motif 1 Motif 1	SARS-CoV-2	156	48.67	8.87	25105	1.288
	SARS-CoV	154	43.09	9.20	24980	1.268
Motif 2 Motif 2	SARS-CoV-2	105.51	55.70	10.69	12490	0.555
	SARS-CoV	101.43	46.95	11.42	12490	0.545
Motif 3 Motif 3	SARS-CoV-2	76.20	24.15	9.52	7450	-0.464
	SARS-CoV	74.20	16.91	9.52	7450	-0.568

Table 3 indicates the comparison of RNA polymerase of SARS-CoV and SARS-CoV-2 in some of their physiochemical properties. According to results mentioned in Table 3, there is no important difference between the values.

**Table 3:** Physicochemical properties of whole protein and most probable motifs of RNA polymerase protein of SARS-CoV-2 and SARS-CoV

**Motif**	**Protein**	**Aliphatic**	**Instability**	**PI**	**Extension Coefficient**	**GRAVY**
-	RNA polymerase SARS-CoV-2	78.43	28.32	6.14	137670	-0.224
-	RNA polymerase SARS-CoV*	80.13	29.36	6.06	137420	-0.170
Motif 1 Motif 1	SARS-CoV-2	70.40	42.61	9.23	8730	-0.25
	SARS-CoV	70.40	44.11	9.23	8730	-0.30
Motif 2 Motif 2	SARS-CoV-2	68.20	30.37	4.98	14440	-0.76
	SARS-CoV	68.20	30.37	4.98	14440	-0.76
Motif 3 Motif 3	SARS-CoV-2	42.80	46.30	4.96	13075	-0.88
	SARS-CoV	42.80	46.30	4.96	13075	-0.88

* The mean value of parameters among four sequences has been reported.

Pairwise alignment of SARS-CoV and SARS-CoV-2 membrane protein sequences revealed 22 mutation positions including one insertion and twenty-one substitution mutations in SARS-CoV. While, twenty-four mutation sites were discovered through SARS-CoV and SARS-CoV-2 RNA polymerase proteins alignment (Table 4). Effect of substitution mutations on stability of protein was analyzed by I-Mutant 2.0 server. In this server, predicted free energy change value (DDG) is calculated via equation 1. DDG values less than zero show stability decrease in the new protein, while positive values for DDG show stability increase. In Table 4, only the sign of DDG, i.e. decrease or increase, is shown.

Free energy change value DDG=DG (Kcal/mol, NewProtein)-DG(Kcal/mol, WildType) Eq.1

Based on I-mutant server, results for membrane protein, all positions (excluding positions 14 and 39) had negative score showing stability decreasing in SARS-CoV-2 protein in comparison to SARS-CoV (Table 4). In case of RNA polymerase, I-mutant analysis outcomes showed that nearly all mutations resulted in stability enhancement in SARS-CoV-2 RNA polymerase. 

**Table 4:** Results of instability and function changes analysis using I-Mutant and SIFT servers for all mutation positions in membrane protein and RNA polymerase protein in SARS-CoV and SARS-CoV-2

**Mutation** **SARS-CoV** → ** SARS-CoV-2**	**I-Mutant Score** **25** ^0^ **C/ Sign of DDG**		**SIFT Score**	**Mutation** **SARS-CoV** → ** SARS-CoV-2**	**I-Mutant Score** **25** ^0^ **C/ Sign of DDG**		**SIFT Score**
**Membrane protein**				**RNA polymerase protein**			
K11E	Decrease		1.00	S5Q	Increase		0.01
Q14K	Increase		1.00	T6S	Increase		0.66
A29T	Decrease		0.07	G63D	Increase		0.00
M32C	Decrease		0.01	L66I	Increase		0.07
S39A	Increase		0.08	M77F	Increase		0.14
V51I	Decrease		1.00	A85T	Increase		0.67
V75I	Decrease		0.07	V90L	Increase		1.00
I86L	Decrease		0.03	V98K	Decrease		0.52
V96I	Decrease		0.16	V225T	Increase		1.00
R124H	Decrease		0.13	A226T	Increase		0.34
V128L	Decrease		0.10	C229S	Increase		0.37
M133L	Decrease		0.32	A252T	Increase		0.09
I144L	Decrease		0.41	M257V	Increase		1.00
M150I	Decrease		0.41	A259T	Increase		0.43
S154H	Decrease		0.24	A262T	Increase		0.62
G187A	Decrease		0.12	L265Y	Increase		1.00
T188G	Decrease		0.19	C281K	Decrease		0.86
N196S	Decrease		0.10	T611N	Increase		1.00
A210S	Decrease		0.74	S643T	Increase		0.73
G211S	Decrease		0.87	N647S	Increase		1.00
N213S	Decrease		0.83	H739T	Increase		0.48
				N769T	Increase		0.32
				A772S	Increase		0.97
				A784S	Increase		0.16

*Deleterious positions (values less than threshold value 0.05) are highlighted in dark gray. Values very close to threshold value are highlighted in light gray.

Function-related influence of mutations was studied by SIFT webserver. The scaled probability values for each mutation are inserted in Table 4. According to SIFT server threshold, value less than 0.05 for a substitution is predicted as deleterious which means the mutation can change function of the protein. In SARS-CoV membrane protein, among 21 substitution mutations, two positions M32C and I86L were predicted as deleterious and three positions A29T, S39A and V75I had values very close to critical value of 0.05. In RNA polymerase protein, mutations S5Q and G63D were deleterious and could cause function changes in the protein. L66I had a close value to the critical value of 0.05 that showed the probability of function changes in this region as well. 

According to N-linked and O-linked glycosylation site analysis using GPP webserver, 17 and 22 glycosylation sites were predicted in SARS-CoV and SARS-CoV-2 membrane protein, respectively. The five new glycosylation sites in SARS-CoV-2 are related to mutation positions 4, 197, 211, 212 and 214, which are mainly due to substitution or insertion of serine (S) in the sequence. For RNA polymerase protein, 59 and 66 glycosylation sites were predicted in SARS-CoV and SARS-CoV-2, respectively. Among new positions, six positions were associated with mutation positions 226, 229, 259, 611, 772 and 784. 

## DISCUSSION

In this study, RNA polymerase and membrane proteins of SARS-CoV and SARS-CoV-2 were compared individually in aspects of their physiochemical properties, mutation positions, and mutation influence on stability and glycosylation patterns. Instability index of membrane protein of SARS-CoV-2 was about 40 which was more than that of SARS-CoV. In literature, it is reported that a protein with instability index smaller than 40 is considered as stable and with above value of 40 is considered as an unstable protein [13]. In this regard, the membrane protein of SARS-CoV-2 has shown more unstable property in its whole protein and motifs in comparison to SARS-CoV [26, 27]. 

The mutation positions analyzed by pairwise alignment, I-Mutant and SIFT webservers, showed 22 and 24 mutations in membrane and RNA polymerase proteins of SARS-CoV-2, respectively. These mutations have resulted in increasing the stability of RNA polymerase but decreasing the stability of membrane protein and cause function changes in the latter protein. According to UniProt webserver, data about topological domains of membrane protein (P0DTC5), mutation positions with function changes (M32C and I86L) are located in transmembrane domain of the protein. Regarding the function of membrane protein, mutation in transmembrane domains can be important and have influence on self-assembly for multimeric structure forming or on anchoring of the protein to the host Golgi membrane [28, 29].

An idea about relationship between stability and function of enzyme proteins indicates that catalytic residues responsible for catalytic function of an enzyme are not optimized for stability. In other words, mutations in active sites resulting in increase of stability cause reduction in enzymatic activity [30, 31]. In case of SARS-CoV-2 RNA polymerase, the majority of mutations are located out of catalytic domain (611-775). This fact could mean that the stability of the RNA polymerase of SARS-CoV-2 has increased and the proteins high catalytic activity has remained, simultaneously. In addition, five new glycosylation sites in SARS-CoV-2 membrane protein were observed which were mainly due to substitution or insertion of serine (S) in the original sequence. It seems that there is a bias toward substitution of amino acids in SARS-CoV membrane protein sequence to serine, making new glycosylation sites in this protein in SARS-CoV-2 and subsequently increasing pathogenicity of the protein. Moreover, seven different glycosylation sites were observed in SARS-CoV-2 RNA polymerase that six positions were corresponding to mutations. It could be interpreted that SARS-CoV-2 increases its RNA polymerase stability not only via DDG positive mutations, but also through providing new positions for more glycosylation.

Another important point that should be considered is host immune system evasion. One of the early mechanisms for recognition of pathogens depends on the glycosylation pattern of a pathogen. Pathogenic proteins with more glycosylation sites can exhibit more similar behavior to the host proteins making recognition process very difficult for host immune system. Moreover, glycosylated sites can act as a cover for the protein that lead to more evasion of pathogen [32]. In case of SARS-CoV-2, increasing in glycosylation sites in both membrane protein and RNA polymerase may result in the protein intracellular niche adaptation and host immune system evasion. 

In overall, substitution and insertion mutations of membrane glycoprotein of SARS-CoV-2 result in more protein instability but provide more glycosylation sites in the protein. More glycosylation of protein can aid the virus to evade from the host immune system [32]. On the other hand, RNA polymerase of SARS-CoV-2 showed more stability than SARS-CoV RNA polymerase and glycosylation analysis predicted more glycosylation sites in this protein, as well. Due to very important role of RNA polymerase in lifecycle of a virus, increasing the number of glycosylation sites and stability of this protein can be assumed as one of the significant reasons of SARS-CoV-2 high pathogenicity. 

## Conflict of Interest

The authors have no conflicts of interest to declare
